# The Capillary Morphogenesis Gene 2 Triggers the Intracellular Hallmarks of Collagen VI-Related Muscular Dystrophy

**DOI:** 10.3390/ijms23147651

**Published:** 2022-07-11

**Authors:** Enrico Castroflorio, Ana Joaquina Pérez Berná, Arístides López-Márquez, Carmen Badosa, Pablo Loza-Alvarez, Mónica Roldán, Cecilia Jiménez-Mallebrera

**Affiliations:** 1 ICFO-The Institute of Photonic Sciences, The Barcelona Institute of Science and Technology, 08860 Castelldefels, Spain; pablo.loza@icfo.eu; 2 ALBA Synchrotron Light Source, 08290 Cerdanyola del Vallès, Spain; anperez@cells.es; 3 Laboratorio de Investigación Aplicada en Enfermedades Neuromusculares, Unidad de Patología Neuromuscular, Servicio de Neuropediatría, Institut de Recerca Sant Joan de Déu, 08950 Esplugues de Llobregat, Spain; aristides.lopez@sjd.es (A.L.-M.); mariacarmen.badosa@sjd.es (C.B.); 4 Institut de Recerca Sant Joan de Déu, 08950 Esplugues de Llobregat, Spain; monica.roldan@sjd.es; 5 Centro de Investigaciones Biomédicas en Red de Enfermedades Rara (CIBERER), 28029 Madrid, Spain; 6 Unitat de Microscòpia Confocal i Imatge Cellular, Servei de Medicina Genètica i Molecular, Institut Pediàtric de Malaties Rares (IPER), Hospital Sant Joan de Déu, 08950 Esplugues de Llobregat, Spain; 7 Department of Genetics, University of Barcelona, 08028 Barcelona, Spain

**Keywords:** collagen VI, CMG2, super-resolution microscopy, muscular dystrophy, patient-derived fibroblasts

## Abstract

Collagen VI-related disorders (COL6-RD) represent a severe form of congenital disease for which there is no treatment. Dominant-negative pathogenic variants in the genes encoding α chains of collagen VI are the main cause of COL6-RD. Here we report that patient-derived fibroblasts carrying a common single nucleotide variant mutation are unable to build the extracellular collagen VI network. This correlates with the intracellular accumulation of endosomes and lysosomes triggered by the increased phosphorylation of the collagen VI receptor CMG2. Notably, using a CRISPR-Cas9 gene-editing tool to silence the dominant-negative mutation in patients’ cells, we rescued the normal extracellular collagen VI network, CMG2 phosphorylation levels, and the accumulation of endosomes and lysosomes. Our findings reveal an unanticipated role of CMG2 in regulating endosomal and lysosomal homeostasis and suggest that mutated collagen VI dysregulates the intracellular environment in fibroblasts in collagen VI-related muscular dystrophy.

## 1. Introduction

Congenital muscular dystrophy (CMD) is a group of rare neuromuscular diseases, which are highly disabling and lead to a reduced life expectancy [1,2]. In particular, common CMD related to collagen VI deficiency (COL6-RD) vary in severity and have no curative treatment.

Collagen type VI is a micro fibrillary macromolecule assembled through a finely regulated process. It is expressed in different tissues such as muscles, tendons, cartilages, blood vessels, and the brain [3,4,5,6], and it is mainly produced by fibroblasts. Once secreted in the extracellular space, collagen VI tetramers associate end-to-end-forming microfibrils that create a network in the interstitial space. Collagen VI has a pivotal role in maintaining skeletal muscle integrity and function allowing a proper link between muscle cells and the extracellular matrix (ECM).

*COL6A1*, *A2*, and *A3* are the genes encoding the three main chains of collagen VI, and mutations in these genes cause the various forms of COL6-RD [7,8,9]. The collagen VI mutations affect both the ECM and the intracellular organelles such as the Golgi apparatus, mitochondria, and the autophagic system, leading to apoptotic events [6,10,11,12]. The connective tissue features of collagen VI deficiency are independent of skeletal muscle dysfunction and are directly related to the role of collagen VI in the organisation and maintenance of the extracellular matrix in the affected tissues [5]. Furthermore, fibroblasts are the main source of collagen VI in skin and muscle and therefore are the primary disease cell type and target [13].

The transmembrane protein capillary morphogenesis gene 2 (CMG2, also known as anthrax toxin receptor 2, ANTXR2) has recently been uncovered as a collagen VI receptor [14]. When in its ligand-bound state, conformational changes of the receptors allow the src-dependent recruitment of the actin cytoskeleton regulator RhoA and its effectors thus inducing receptor endocytosis [15,16]. However, the role of CMG2 in the collagen VI-dependent regulatory processes remains controversial.

In this work, we reveal a direct link between collagen VI mutation and CMG2 activity. Our super-resolution microscopy and soft X-ray tomography analyses show that COL6-RD patient-derived fibroblasts are unable to form the typical extracellular collagen VI matrix. Moreover, mutant cells show the characteristic intracellular hallmark of the pathology, such as mitochondria and Golgi fragmentation. Using advanced microscopy techniques, we highlighted a novel intracellular impairment characterised by endo-lysosomal system accumulation. Because of the tight association between the CMG2 receptor and the endocytic pathways [15,16], we biochemically analysed CMG2 revealing an increase in phosphorylation levels in patient-derived fibroblasts.

The dominant variants account for 50–75% of all pathogenic variants in COL6-RDs [17]. Using a gene-editing tool able to silence the single nucleotide variant mutation in the *COL6A1* gene (COL6A1 c.877G>A; p.Gly293Arg) in patients’ cells, we were able to restore all the pathological phenotypes observed.

Thus, given the known association of the collagen VI Gly293Arg substitution mutation variant in congenital muscular dystrophy and the lack of functional knowledge regarding the effects of this mutation on intracellular homeostasis, here we generated a new cellular model based on the use of super-resolution and advanced microscopy to investigate the significance of collagen VI mutations for the intracellular environment.

## 2. Results

### 2.1. Patient-Derived Fibroblasts Cannot Assemble the Extracellular Collagen VI Network

**Figure 1:**
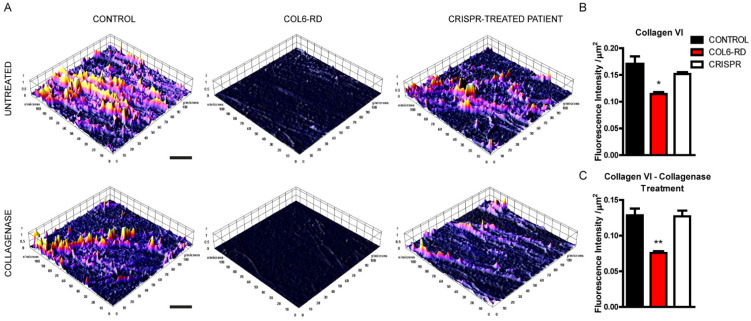
Altered extracellular matrix in cultured COL6-RD patient-derived fibroblasts. (**A**) Representative 3D surface plot generated from the STED images (Figure S1) of the extracellular collagen VI matrix in untreated (upper panel) and collagenase-treated (lower panel) controls, patients, and CRISPR-treated fibroblasts showing the COL6 fluorescence levels. Fluorescence intensity analysis was also quantified in untreated (**B**) and collagenase-treated (**C**) samples. Data are expressed as the mean ± SEM; *n* = 3; independent primary cell preparations (30 images of 8–10 stacks for each CONTROL, COL6-RD, and CRISPR samples) were used for all panels. * *p* < 0.05, ** *p* < 0.01; one-way ANOVA/Bonferroni’s multiple comparison test. Scale bar 5 µm.

To assess the structure of collagen VI fibres with high resolution, we performed STED microscopy in control and COL6-RD patient-derived fibroblasts. First, we assessed the endogenous extracellular collagen VI matrix revealing a severely affected fibre structure in COL6-RD patient-derived fibroblasts. The 3D surface plot of the STED images (Figure S1) shown in Figure 1A (upper panel), highlights how the control extracellular collagen VI matrix is formed by fibres that may or may not have specific orientations in cell cultures. On the contrary, patient-derived fibroblasts are unable to build collagen VI fibres resulting in amorphous and blob-like extracellular components. Moreover, to determine the stability and resistance of the blobby mutant collagen VI matrix, we treated control and patient-derived fibroblasts with collagenase, a protease able to degrade the triple-helical native collagen fibrils [18]. As shown in the lower panel of Figure 1A, collagenase treatment completely degraded the mutant extracellular collagen VI secreted by COL6-RD patient-derived fibroblasts. On the contrary, the control matrix is only mildly affected and still reveals collagen VI fibrils (Figure 1B).

Given the impact of COL6-RD on patients and families, there is a pressing need for newer and less-invasive therapies. To address this clinical need, we recently described a gene-editing technique based on CRISPR-Cas9 to silence the Gly293Arg substitution in human dermal fibroblasts [19]. We used the same strategy to test whether the restoration of the mutation in COL6-RD patient-derived fibroblasts would be sufficient to allow cells to recover the ability to build a collagen VI extracellular network. Crucially, the therapy was sufficient to rescue the extracellular matrix phenotypes to control values for both untreated and collagenase-treated mutant cells (Figure 1A–C). These data indicate that the gene-editing treatment can re-establish the ability to shape the normal collagen VI networks in patient-derived fibroblasts.

### 2.2. The Endo-Lysosomal System Homeostasis Is Altered in COL6-RD Patient-Derived Fibroblasts

As previously shown, collagen VI mutations affect the intracellular homeostasis in patient-derived fibroblasts and myoblasts [10,14,20]. Using STED microscopy, we were able to detect both mitochondria and Golgi apparatus fragmentation (stained with TOM20 and GM130 antibodies, respectively), revealing that patient-derived fibroblasts have altered the morphology of these organelles [10,21,22] (Figures S2 and S3). To further characterise the intracellular environment of patient-derived fibroblasts, we used cryo-soft X-ray tomography (Cryo-SXT). This synchrotron-based technique uses soft X-rays to image samples in the water-window energy absorption range (520 eV) [23,24]. Cryo-SXT is the only available imaging technique that can yield nanometer-resolution 3D maps from vitrified whole-cell samples (thus avoiding chemical treatment or sectioning of the sample and the potential artefacts that come with these treatments). Using Cryo-SXT on COL6-RD patient-derived and control fibroblasts, we examined ∼10 cell 3D volumes under each condition and revealed an accumulation of mitochondria, endosome/lysosome-like structures, and multivesicular bodies (MVB, Videos S1–S3). In Figure S4, several endocytic vesicles are shown. Their classification depends on the morphology described in fibroblasts [25]. Based on their morphology alone, we have classified the vesicles into two groups: endosome/lysosome-like vesicles in violet, and MVB in pink (Figure S4). The vesicles with cup-shaped high absorbing form on one side of the vesicle shown in Figure S4A,B,E,F,J,K represent the typical morphology of endosomes and lysosomes. MVB can have different appearances although they are easily recognized by their usually high absorbing content with one or multiple low-absorbing vesicles within (Figure S4C,D,G,H,L,M). We did not detect morphological differences in either endosome/lysosome-like structures or multivesicular bodies between the controls, COL6-RD patients, or CRISPR-treated fibroblasts.

**Figure 2:**
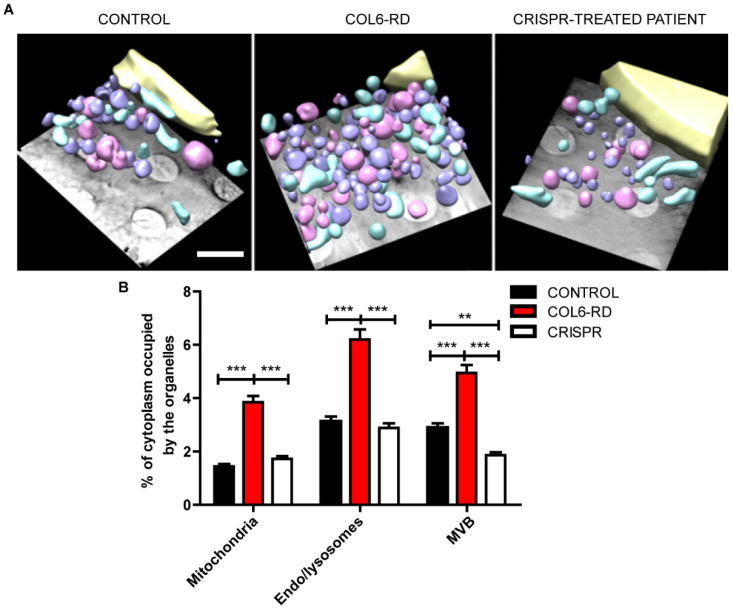
Altered abundance of mitochondria, endo/lysosomal-like vesicles, and multivesicular bodies in COL6-RD patients’ cells. (**A**)Three-dimensional reconstruction of whole-cell volumes of control-, andCOL6-RD patient-derived fibroblasts and CRISPR-treated fibroblasts. Threshold-based isosurface segmentation of the surface boundaries identifies the different organelles present in the cells: nucleus in yellow, mitochondria in light blue, endo/lysosomal-like vesicles in violet, and multivesicular bodies in pink. (**B**) Quantification of the percentage of cytoplasm occupied by the organelles analysed. Data are expressed as the mean ± SEM; *n* = 10; different cells (10 CONTROL cells, 10 COL6-RD cells, and 10 CRISPR samples) were used for all panels. ** *p* < 0.01, *** *p* < 0.001; Two-way ANOVA/Bonferroni’s multiple comparison test. Scale bar 500 nm.

Nevertheless, using Cryo-SXT, we highlighted the differences between control and COL6-RD patient-derived fibroblasts in the quantification of mitochondria, endosome/lysosome-like vesicles, and MVB expressed as the percentage of cytoplasm volume occupied (Figure 2A). Interestingly, the absence of the normal collagen VI protein induced a ∼twofold increase in cytoplasm volume occupied by mitochondria, endo/lysosome-like structures, and MVB in the COL6-RD patient-derived fibroblasts compared to the control (Figure 2B). Crucially, after CRISPR treatment, the abundance of mitochondria, endosomes, and lysosomes returned to the control levels (Figure 2B). Surprisingly, the percentage of cytoplasm occupied by MVB after the CRISPR treatment decreased to below the control level (Figure 2B). In summary, these data suggest that the density of the cytoplasmic structures analysed is upregulated in COL6-RD patient-derived fibroblasts and that CRISPR treatment can rescue the pathological phenotype of *COL6A1* mutated cells.

**Figure 3:**
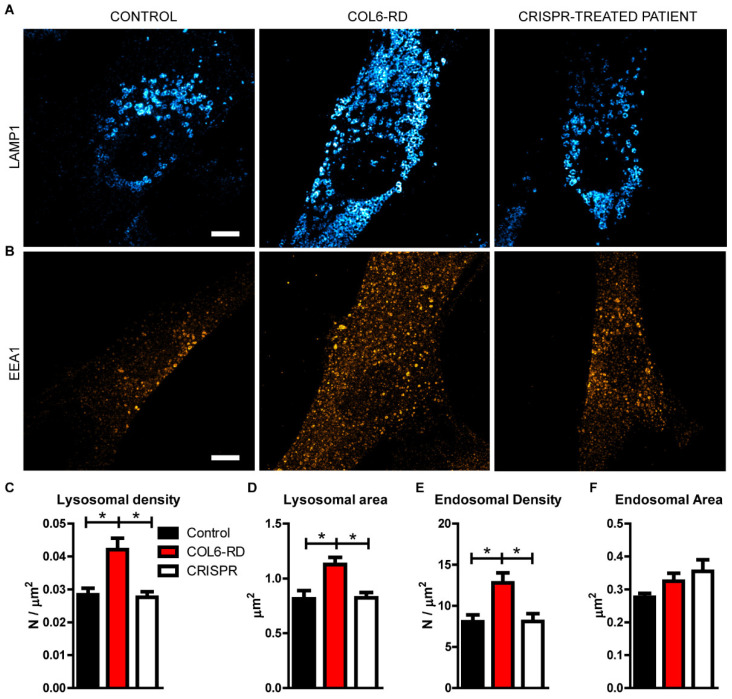
The endo-lysosomal compartments are altered in COL6-RD patient-derived fibroblasts. (**A**) Representative STED images of Lamp1- and (**B**) EEA1-positive organelles labelled in control- and COL6-RD patient-derived fibroblasts and in CRISPR-treated COL6-RD patient-derived fibroblasts. (**C**) Lamp1- and (**E**) EEA1-positive organelles density, together with the area of (**D**) Lamp1- and (**F**) EEA1-positive organelles was quantified. Data are expressed as the mean ± SEM; *n* = 3; independent primary cell preparations were used for all panels. * *p* < 0.05; one-way ANOVA/Bonferroni’s multiple comparison test. Scale bar 5 µm.

With the aim of characterising the vesicular organelle accumulation in collagen VI-mutated human fibroblasts observed using Cryo-SXT, we labelled the endo-lysosomal system and applied super-resolution microscopy. Using STED microscopy, we detected an accumulation of lysosomes, labelled with the lysosomal-associated membrane protein 1 (Lamp1) antibody in patient-derived fibroblasts, compared to the control ones (Figure 3A–C). Furthermore, we detected a bigger lysosomal area in COL6-RD patient-derived fibroblasts (Figure 3D). Intriguingly, we also found an alteration of the upstream endocytic pathway labelling the early endosome antigen 1 (EEA1)-positive vesicles in COL6-RD samples with respect to the controls (Figure 3B,D–F).

**Figure 4:**
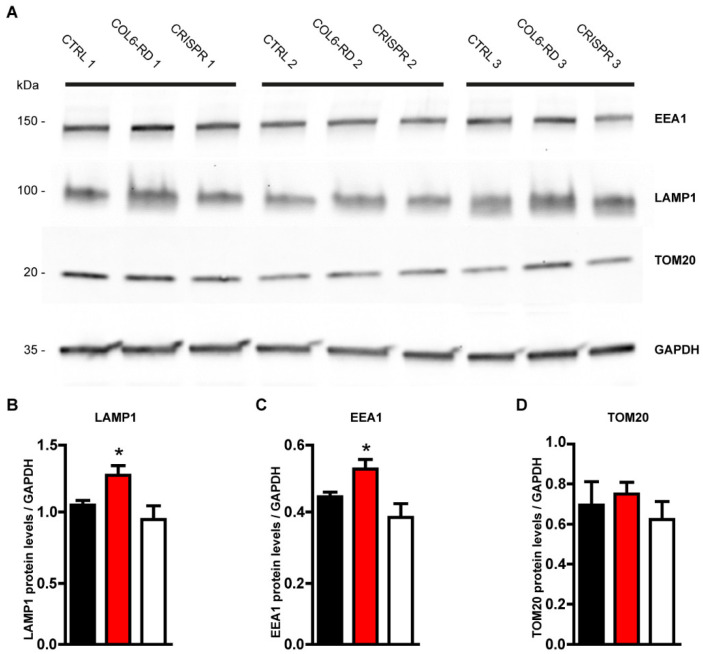
Biochemical confirmation of the intracellular phenotypes observed with super-resolution microscopy. (**A**) Primary fibroblast homogenates from control and COL6-RD patients and CRISPR-treated COL6-RD patient-derived fibroblasts were subjected to Western blotting using antibodies against Lamp1, EEA1, and Tom20 as shown. The GAPDH marker was used as the loading control. (**B**) Quantification of Lamp1 from the three phenotypes. (**C**) Total quantification of EEA1 from the three phenotypes. (**D**) Total quantification of Tom20 from all the samples. Data are expressed as the mean ± SEM; *n* = 3; independent primary cell preparations were used for all panels. * *p* < 0.05; one-way ANOVA/Bonferroni’s multiple comparison test.

Given the abundance of endosomes and lysosomes and the fragmentation of mitochondria observed with both the STED and Cryo-SXT techniques, we examined the protein levels of the endosomal, lysosomal, and mitochondrial markers from the control and patients’ fibroblast extracts. The lysosomal marker Lamp1, together with the endosomal marker EEA1, were significantly increased as observed by Western blot analysis (Figure 4A–C). On the contrary, the mitochondrial marker TOM20 was unaffected (Figure 4A,D). These data indicate that the accumulation of the endolysosomal system detected with both advanced microscopy and Western blot reflects the real cytoplasmic abundance of these organelles in COL6-RD patient-derived fibroblasts. However, the abundance of mitochondria detected with Cryo-SXT microscopy does not reflect the real cytoplasmic abundance of this organelle but its fragmentation, as detected with super-resolution microscopy.

Gene editing based on the CRISPR-Cas9 technique was also remarkably effective for all the intracellular pathological phenotypes described. We were able to restore mitochondria and Golgi fragmentation (Figures S2 and S3), and the endolysosomal accumulation (Figure 2, Figure 3 and Figure 4), reaching a non pathological-like phenotype in all the COL6-RD patient’s cells that we treated and analysed.

Altogether, these data indicate that the collagen VI mutations directly affect the homeostasis of different intracellular compartments.

### 2.3. Col VI Mutation Induces Hyper-Phosphorylation of the CMG2 Receptor in Humans

Capillary morphogenesis gene 2 (CMG2) encodes a single-pass transmembrane protein harbouring an extracellular von Willebrand A (vWA) domain proposed to bind collagen VI [14]. Furthermore, it has been shown that collagen VI-dependent CMG2 activation triggers the phosphorylation of its tyrosine site, signalling the cells to start membrane retrieval events [16]. Therefore, in view of the altered extracellular matrix and the intracellular endolysosomal accumulation observed, we questioned whether the binding activity of the mutated collagen VI to the CMG2 receptor would be modified in patient fibroblasts.

**Figure 5:**
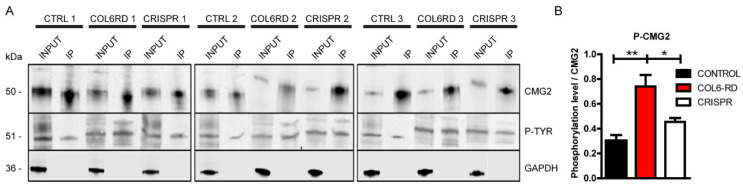
CMG2 phosphorylation levels are increased in COL6-RD patient-derived fibroblasts. (**A**) Western blotting for CMG2 receptor and its tyrosine-phosphorylated form after immunoprecipitation (IP) of control, COL6-RD patient, and CRISPR-treated COL6-RD patient-derived fibroblasts. Blots of equally loaded protein extracts prior to IP (input) are also shown. The marker GAPDH is used as the loading control. (**B**) Total quantification of the IP phosphorylated form of CMG2 versus IP CMG2 from the three phenotypes is shown. Data are expressed as the mean ± SEM; *n* = 3; independent primary cell preparations were used for all panels. * *p* < 0.05, ** *p* < 0.01; one-way ANOVA/Bonferroni’s multiple comparison test.

First, we assessed the endogenous production of CMG2 in control and patient-derived fibroblasts through Western Blot and STED microscopy, revealing no differences between the two genotypes in terms of protein production (Figure S5A,B) and membrane localisation (Figure S5C–E). Next, to analyse the CMG2 phosphorylation levels without an available antibody able to recognize the phosphorylated form of the receptor, we performed immunoprecipitation experiments from human fibroblast lysates using an anti-CMG2 antibody. We then analysed the phosphorylation of the receptor in the immunoprecipitated content using a phospho-tyrosine antibody (Figure 5A). Western blot analysis revealed increased phosphorylation levels of the CMG2 receptor in COL6-RD patient-derived fibroblasts compared to the controls. Importantly, CRISPR-mediated silencing of the dominant-negative mutation reduced the CMG2 receptor phosphorylation levels of COL6-RD patient-derived cells, bringing the levels close to those observed in the control samples (Figure 5A,B).

These data revealed that the CMG2 receptor is the link between the mutated extracellular collagen VI and the intracellular endo-lysosomal accumulation.

## 3. Discussion

Here, we demonstrate for the first time the link between the extra- and the intracellular alterations caused by *COL6A1* heterozygous c.877 G>A; p.Gly293Arg nucleotide substitution in COL6-RD patient-derived fibroblasts. We show (i) extracellular collagen VI network abnormalities in terms of morphology and enzyme digestion resistance and (ii) a stronger activation of the collagen VI receptor CMG2 accompanied by (iii) endo-lysosomal system accumulation, and (vi) both mitochondria and Golgi apparatus fragmentation in primary human fibroblasts. Importantly, we could rescue the extracellular collagen VI network, the CMG2 phosphorylation levels, and the intracellular alterations by CRISPR-mediated silencing of the *COL6A1* mutation.

Our extracellular collagen VI characterisation study made with STED microscopy points towards a highly unorganised collagen network. Anatomically, collagen VI is distributed in the stromal interface surrounding interstitial cells in proximity to the basement membrane [3]. Once secreted in the extracellular space, tetramers associate end-to-end forming microfibrils that create a network able to maintain skeletal muscle function and integrity and allow a proper link between muscle cells and the extracellular matrix (ECM) [12,13,23]. Importantly, the Gly293Arg substitution introduces folds/kinks that prevent proper assembly with other tetramers to form collagen VI microfibrils [8,26,27]. This conformational change further exposes the triple-helical domains of the α-chains (characterised by the Gly-X-Y motif, where Gly is Glycine, X is Proline, and Y is Hydroxyproline or Hydroxylysine) to the enzyme collagenase that can now easily localise and hydrolyse the bond between Gly-X-Y [28,29,30].

We identified a significant shift in the collagen VI receptor CMG2 phosphorylation levels. Being unable to build the proper collagen VI network, the mutated molecules are dispersed in the extracellular matrix, hence more prone to bind to receptors on the cell membranes. As previously shown in [31], collagen VI is digested by fibroblasts through the lysosomal system after the endocytosis of the plasma membrane. Importantly, extracellular proteases are acting on collagen VI even before the actual internalisation to trigger the endocytic process [31]. In this regard, we showed that mutated collagen VI is more sensitive to extracellular proteases such as collagenase. Thus, given that the dominant mutation studied in this work alters the structure of collagen VI molecules, it is possible that mutated collagen VI binds to the CMG2 receptor more easily than wild-type collagen VI.

We did not detect changes in the absolute levels of CMG2 in patient-derived fibroblasts, either with Western Blot or super-resolution microscopy. These data are consistent with previous studies showing that CMG2 activation does not affect CMG2 expression but does disturb cell homeostasis [15,16]. Of note, CMG2 loss-of-function mutations have also been associated with hyaline fibromatosis syndrome, where collagen VI accumulates massively in the uterus of CMG2^-/-^ mice and leads to progressive fibrosis and sterility without displaying changes in collagen gene expression [14]. Furthermore, mutations in the CMG2 gene also lead to infantile systemic hyalinosis, causing diffuse deposition of hyaline material—including collagen—in the skin, gastrointestinal tract, muscles, and endocrine glands, thus altering intra- and extracellular environments [32]. Therefore, our data provide further evidence for the importance of CMG2 modulation for cellular homeostasis.

We further demonstrate that the collagen VI single nucleotide variant analysed in this study triggers an accumulation of endosomes and lysosomes that was previously uncharacterised. Using STED microscopy coupled with cryo-soft X-ray tomography (Cryo-SXT)—a synchrotron-based technique that allows nanometer-resolution 3D maps of a sample [33]—we detected an increased density of endosome- and lysosome-like structures per cell area. Intriguingly, CRISPR-mediated silencing of the dominant-negative mutation rescued the mitochondria, endosomes, and lysosome accumulation. The use of the gene-editing tool on COL6-RD patient-derived fibroblasts revealed an exaggerated reduction of MVBs. In the endocytic pathway, MVBs are late endosomes whose content can be degraded through fusion with lysosomes [34,35,36]. Thus, it is possible that the massive reduction in endosomes and lysosomes triggered by the CRISPR-mediated treatment caused the excessive reduction of MVBs.

Evidences showed that the endolysosomal compartment is pivotal in modulating the extracellular matrix environment by regulating endocytic trafficking [37,38,39,40,41,42]. The endolysosomal alteration we found, coupled with the higher phosphorylation levels of CMG2, is consistent with the receptor physiology; as elegantly described by Bürgi et al., CMG2-extracellular ligand (collagen VI) binding leads to proto-oncogene tyrosine-protein kinase src-dependent talin release and recruitment of the actin cytoskeleton regulator RhoA and its effectors. CMG2 can now be phosphorylated and internalised for intracellular degradation [16]. In addition, mutant cells exhibited altered mitochondria morphology, a key intracellular hallmark of COL6-RD pathology [6,10]. Our observation of the mitochondrial alterations is coherent with studies made in myofibers of Col6a1^-/-^ mice revealing mitochondrial dysfunction and spontaneous apoptosis [10]. Also, clinical studies indicated that cyclosporin A may help to slow COL6-RD progression by correcting the mitochondrial dysfunction [43,44,45]. In addition, single-cell (sc) RNA-sequencing analysis demonstrated that mitochondrial respiratory chain dysfunction is a key factor that can alter ECM integrity and mechanostability [46]. We also detected Golgi apparatus fragmentation and morphological modifications in COL6-RD patient-derived fibroblasts. Golgi fragmentation occurs in pathological situations, such as when apoptosis is activated and when vesicular secretory trafficking is perturbed [21,47,48,49,50,51,52]. Furthermore, several evidences show that the Golgi apparatus plays a key role in sensing and integrating external and intracellular cues to promote cellular homeostasis [53,54]. Similarly, from what we observed, mice lacking the fukutin gene (Fktn), the causative gene of Fukuyama muscular dystrophy, show myocyte contractile dysfunction and a disordered Golgi network [55]. These studies underline the importance of the altered intracellular organelle homeostasis in muscular pathologies and demonstrate that the ECM is an important regulator of the cytosolic environment. Indeed, Golgi fragmentation was observed in primary fibroblasts of patients affected by distal hereditary motor neuropathy (HMN) and axonal Charcot–Marie–Tooth neuropathy (CMT2) characterised by motor neuron degeneration and distal weakness [56].

Notably, all the results we obtained with STED microscopy were confirmed using Western blotting techniques. On the other hand, Tom20 protein abundance was not modified between controls, patients, and treated patients. These data indicate that mitochondria are morphologically affected and fragmented in patient-derived fibroblasts, as observed with super-resolution microscopy. However, their total number does not change in this pathological condition.

In summary, heterozygous G-to-A substitution at position 877 in exon 10 of *COL6A1* triggers the production of an altered extracellular collagen VI network that subsequently triggers the phosphorylation of the collagen VI receptor CMG2, an accumulation of endosomes and lysosomes and altered mitochondria and Golgi apparatus morphology in human fibroblasts. It has become clear that the ECM has profound effects on many cellular processes [57,58,59,60,61,62,63,64]. Consequently, this work exploits the opportunity to provide clinicians with novel diagnostic tools for monitoring and treating neuromuscular disorders. In the future, it will be important to determine the efficacy of the gene-editing system in vivo to assess important clinical translation outcomes such as delivery and efficacy.

## 4. Materials and Methods

The present study was performed in accordance with the Declaration of Helsinki. Written informed consent was obtained from patients and/or their parents or guardians. Biological samples were stored and managed by the Hospital Sant Joan de Déu (HSJD) Biobank. All experimental protocols were approved by Fundació Sant Joan de Déu Ethics Committee on Clinical Research (CEIC). Methods were carried out in accordance with the relevant guidelines and regulations.

### 4.1. Human Fibroblasts

COL6-RD patients with the same confirmed mutation (*COL6A1* het. c.877G>A, p.G293R) have been described previously [19]. Skin biopsies from the forearm were obtained from COL6-RD patients and children not affected by a neuromuscular condition. For all the experiments, we included 3 COL6-RD patients and 3 age-matched control fibroblast cell lines obtained from the HSJD Biobank. Fibroblasts were grown in Dulbecco’s modified Eagle’s medium (DMEM) with 10% fetal bovine serum (FBS), 1x penicillin/streptomycin, and 1x glutamine (all from Gibco, Waltham, MA) at 37 °C with 5% CO_2_. Confluent fibroblasts (passages 2 or 3) were treated with 25 µg/mL of L-ascorbic acid phosphate magnesium (Wako Chemicals GmbH, Neuss, Germany) for 24 h before the planned experiment. Mild collagenase treatment: collagenase (Type II, Merck) was dissolved in DMEM with a final concentration of 0.02 mg/mL, and confluent fibroblasts were incubated with the enzyme for 10′ at 37 °C with 5% CO_2_.

### 4.2. CRISPR-Cas9

Two crRNAs (crRNA1 and crRNA2) were designed adjacent to the PAM site of the *COL6A1* locus using the Breaking-Cas web tool [65]. The 20 nt-long specific sequences for targeting the *COL6A1* gene were 5′-CCTGGTACCCAACAGGTCTG-3′ (crRNA1) and 5′-CCCGGGGACCTCAGACCTGT-3′ (crRNA2). An 80 nt-long ssDNA donor template was designed containing the wild-type COL6A1 sequence and two silent changes to eliminate the PAM sequence. The sequence of this ssDNA oligo was as follows: 5′-ACCATCTCCTCCTGTGTTCCAGGGAAGACCCGGGGATCTAGGACCTGTTGGGTACCAGGGAATGAAGGTACGTGCCCCCC-3. These reagents and the Alt-R tracrRNA-ATTO550 were synthesised by Integrated DNA Technologies (IDT, Coralville, IA, USA). crRNAs and tracrRNA-ATTO550 reagents were resuspended at 100 μM in 1× Tris-EDTA (TE buffer), pH 8, solution (IDT). Lipofectamine™ CRISPRMax™ (ThermoFisher Scientific, Waltham, MA, USA) was used for the transfection of RNP complexes. First, guide RNA complexes were formed by mixing the crRNA and tracrRNA-ATTO550 in equal molar amounts in IDT Duplex Buffer (30 mM HEPES, pH 7.5, 100 mM potassium acetate) at 1 μM by heating the oligonucleotides to 95 °C and gradually cooling to room temperature. RNP-crRNA mix was prepared in OptiMEM^®^ (Gibco, Waltham, MA, USA) containing the RNA duplex (1 µM), HiFi Cas9 (1 µM), and HiFi Cas9 Plus Reagent (both from ThermoFisher Scientific). The solution was incubated for 5 min at room temperature. The ssDNAs (100 µM) were diluted to a concentration of 1 µM in TE Buffer. Then, a transfection mix of each guide was prepared in duplicate in OptiMEM^®^: 1 µL ssDNA at 1 µM, 200 µL of RNP at 1 µM, and 9.6 µL Lipofectamine™ CRISPRMax™. The final mixture was incubated for 20 min at room temperature. Next, a cell suspension of 400,000 cells/mL in DMEM without antibiotics was prepared. For each volume (800 µL in a 12-multiwell plate) of cell suspension assigned for each guide, 400 µL of the corresponding RNP-ssDNA mixture was added. Finally, CRISPR-transfected fibroblasts were cultured for 24 h at 37 °C with 5% CO_2_ before changing the culture medium and analysing.

### 4.3. Immunofluorescence and Stimulated Emission Depletion (STED) Microscopy

The following primary antibodies were used: mouse anti-collagen VI (1:500, Millipore), mouse anti-Lamp1 (1:100, Abcam, Cambridge, UK), rabbit anti-EEA1 (1:100, Abcam), mouse anti-TOM20 (1:100, Abcam), rabbit anti-GM130 (1:100, ThermoFisher, Waltham, MA, USA), and goat anti-CMG2 (1:100, R&D Systems, Minneapolis, MN, USA). Prior to STED imaging, samples were examined under a Leica CTR5000 fluorescence microscope (Leica) to ensure that at least 90% of the cells were correctly labelled. Fluorescence immunolabeling was performed as previously described [66] with a few modifications. The two-colour labelled samples were observed using a commercial gated-STED microscope (Leica TCS SP8 STED 3X, Leica Microsystem GmbH, Mannheim, Germany) equipped with a pulsed white light laser source and three depletion lasers (592 nm, 660 nm, 775 nm). STED illumination of secondary antibodies coupled with the fluorophore ATTO 647N was performed using a 647 nm line and depletion using a 775 nm line. For the secondary antibodies coupled with the fluorophore Abberior Star 488, the illumination line was 488 nm and the depletion source was 592 nm. Fluorescent light was collected using high-efficiency single-molecule detectors (SMD-HyD) using an HC PL APO CS2 100x/1.40 oil objective. The selected areas were scanned at 600 Hz and the final pixel size was 20 nm. The typical transversal resolution achieved with this system is 80 nm. To study the targeted organelles and proteins in the three dimensions, ten Z stacks were acquired every 0.12 µm along with the cell thickness. To compare the data, identical settings were used for image acquisition in different experiments. The images were deconvolved using the Lightning GPU-based Deconvolution Leica package. All the parameters were analysed using Fiji ImageJ software (National Institutes of Health, Bethesda, MD, USA). The fluorescence intensity of the different dyes was measured as mean intensity and expressed in arbitrary units of fluorescence on the cell area. Images for publication were processed and prepared using Fiji ImageJ software.

### 4.4. Soft X-ray Cryo-Tomography

Sample preparation was performed in the cell culture laboratory at ALBA, where the cells were seeded on gold quantifoil (R2/2) holey carbon grids (Au-G200F1) and incubated for 24 h at 37 °C with 5% CO_2_. Samples were vitrified by plunge-freezing in a Leica EM-CPC. The frozen grids were imaged using a LINKAM CMS196 stage in a Zeiss AxioScope fluorescence microscope. The frozen grids were transferred to a Mistral (ALBA-Light Source, Barcelona, Spain) [67,68] beamline at ALBA synchrotron under cryogenic conditions. The photon energy was set within the water window (520 eV) to take advantage of the high natural absorption contrast of the biological material to acquire X-ray tomography datasets under the conditions described previously [69]. The dataset was acquired using a zone plate objective with an outermost zone width of 40 nm. The effective pixel size in the images was 10 nm. The image stacks were pre-processed to normalise and correct the intensity distribution delivered to the sample by the capillary condenser lens. Alignment of the tilted series was conducted with IMOD [70] and the final reconstructions were made using the iterative SIRT reconstruction option in TOMO3D [71,72]. To enhance the signal-to-noise ratio, TOMOEED was used. Visualisation and manual segmentation (i.e., segmentation of the surface boundaries identifying different organelles to colour-code them) of the final volumes were carried out with AMIRA (Thermo Fisher Scientific, Waltham, MA, USA) and Chimera [73].

### 4.5. Western Blotting and Immunoprecipitation

Primary human fibroblasts were placed on ice and washed twice with ice-cold PBS. Cells were scraped into homogenisation buffer (250 mM sucrose, 1 mM EDTA, 10 mM PMSF, 2 µg/mL aprotinin, 5 µg/mL leupeptin, and 1 µg/mL pepstatin). CMG2 was immunoprecipitated from cell lysates using the Pierce Crosslink Immunoprecipitation Kit (Thermo Fisher Scientific) as specified by the manufacturer. Briefly, antibodies were coupled to the Pierce Protein A/G Plus Agarose for 1 h and then cross-linked to the resin using the DSS crosslinker (2.5 mM in DMSO) for 1 h. Subsequently, 500–1000 µg of lysates was incubated on the column overnight at 4 °C. The eluates were finally loaded on Bis-Tris 4–12% polyacrylamide precast gel and analysed for the presence of the antigen. Blotted membranes were blocked for 1 h in 4% milk or BSA in Tris-buffered saline (10 mM Tris, 150 mM NaCl, pH 8.0) plus 0.1% Tween, and incubated overnight at 4 °C with the following primary antibodies: rabbit anti-EEA1 (1:1000, Abcam), mouse anti-LAMP1 (1:1000, Abcam), mouse anti-TOM20 (1:1000, Abcam), mouse anti-GAPDH (1:5000, ProteinTech, Rosemont, IL, USA), mouse anti-Tubulin (1:5000, Abcam), goat anti-CMG2 (1:1000, R&D systems), and mouse anti-phospho-tyrosine (1:1000, Millipore, Burlington, MA, USA). Membranes were washed and incubated for 1 h at room temperature with peroxidase-conjugated secondary antibodies (1:10.000, Bio-Rad, Hercules, CA, USA). Bands were revealed with the ECL chemiluminescence detection system (Thermo Scientific, Waltham, MA, USA) using the iBright detection machine (Invitrogen) and analysed using Fiji ImageJ software.

### 4.6. Statistical Analysis

Data are expressed as the mean +/− SEM throughout. To compare two normally distributed sample groups, the two-tailed Student’s *t*-test was used. In the case of more than two normally distributed experimental groups, one- or two-way ANOVA followed by multiple comparison tests were employed as stated. The significance level was preset to *p* < 0.05. Data were analysed using Prism 6.0 software (GraphPad Software, Inc., San Diego, CA, USA).

## Data Availability

The data that supported the findings of the present study are available from the corresponding author upon request.

## Supplementary Materials

The following supporting information can be downloaded at: https://www.mdpi.com/article/10.3390/ijms23147651/s1.
