# Supplementary material for: The Capillary Morphogenesis Gene 2 Triggers the Intracellular Hallmarks of Collagen VI-Related Muscular Dystrophy
Source: Int J Mol Sci. 2022 Jul 11;23(14):7651. doi: 10.3390/ijms23147651 (PMC9322809; doi:10.3390/ijms23147651)
Supplement: Supplementary file 1 [file ijms-23-07651-s001.zip › ijms-1796053-supplementary/supplementary figures.pdf]

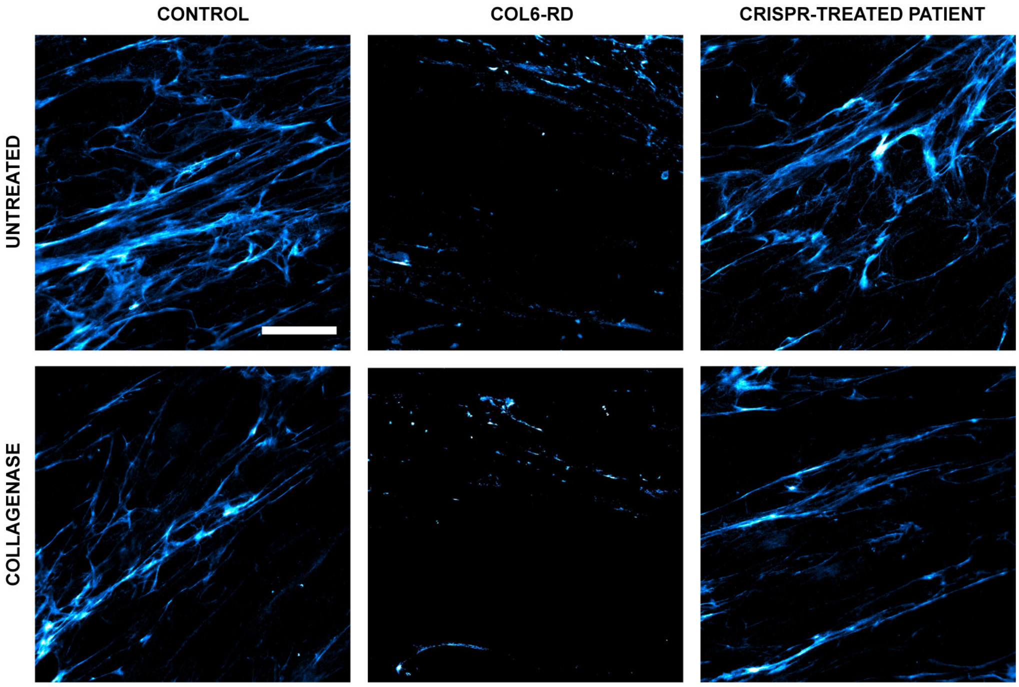

Figure S1. STED imaging of extracellular collagen VI. Representative STED images of the extracellular collagen VI matrix in untreated (upper panel) and mild collagenase-treated (lower panel) fibroblasts derived from control and COL6-RD patient, and CRISPR-treated COL6-RD patient-derived fibroblasts. Scale bar 5 μm.

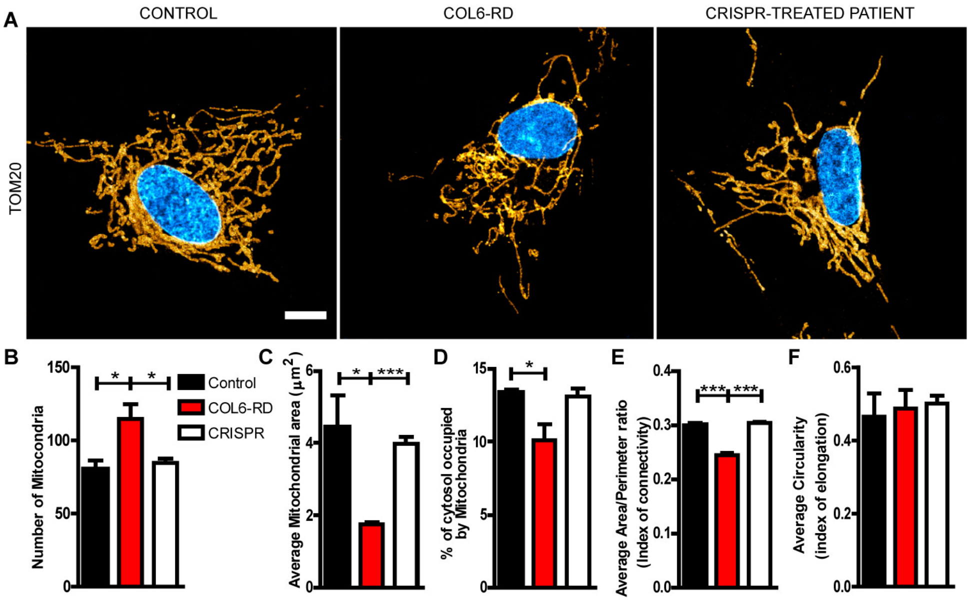

Figure S2. Mitochondrial morphology and content imbalance in COL6-RD patient-derived fibroblasts. (**A**) Representative STED images of control and COL6-RD patient-derived fibroblasts, and CRISPR-treated COL6-RD patient-derived fibroblasts, labelled with Tom-20 (yellow) and dapi for the nuclear staining (blue). Scale bar 5 μm. (**B**) The number of Tom-20-positive organelles, (**C**) the average mitochondrial area, (**D**) the mitochondrial content of the cytoplasm, (**E**) the index of connectivity and (**F**) elongation of the mitochondria was quantified. Data are expressed as the mean ± SEM. $n = 3$ independent primary cell preparations were used for all panels. *$p < 0.05$, **$p < 0.01$, ***$p < 0.001$; one-way ANOVA/Bonferroni's multiple comparison test.

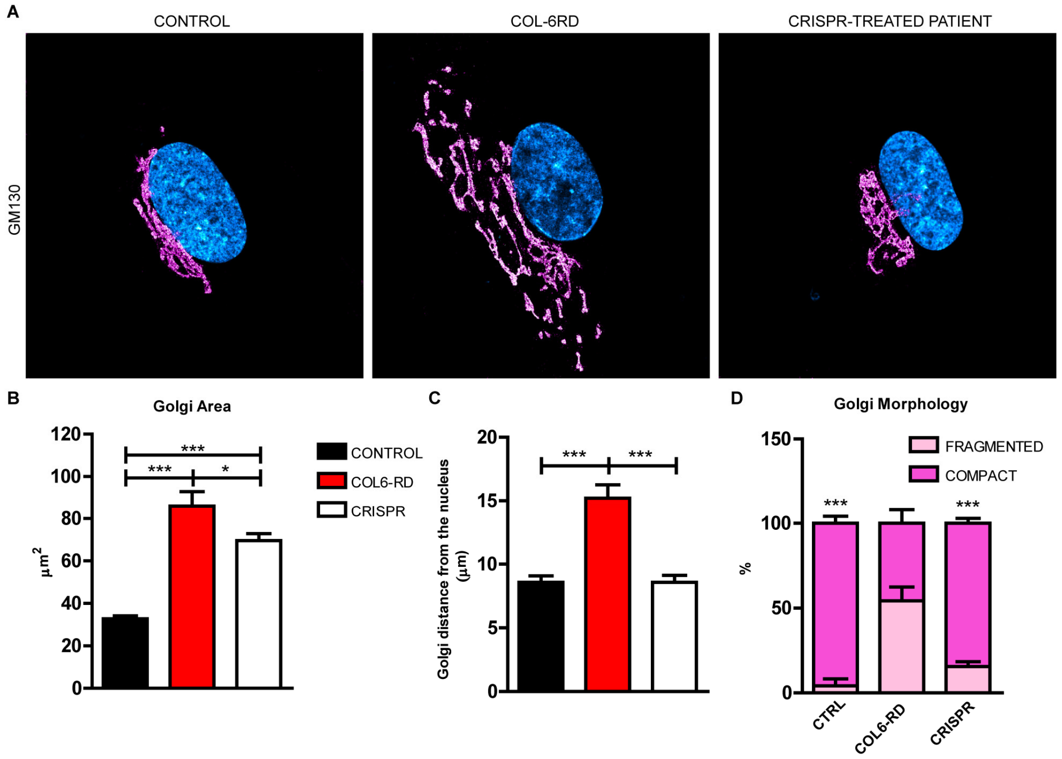

Figure S3. COL6-RD patient-derived fibroblast show fragmented Golgi apparatus morphology. (**A**) Representative STED images of control and COL6-RD patient-derived fibroblasts, and CRISPR-treated COL6-RD patient-derived fibroblasts, labelled with Gm130 (magenta) and dapi for the nuclear staining (blue). Scale bar 5 μm. The Golgi apparatus's area (**B**), distance from the nucleus (**C**), and fragmentation levels (**D**) were quantified. Data are expressed as the mean ± SEM. $n = 3$ independent primary cell preparations were used for all panels. *$p < 0.05$, **$p < 0.01$, ***$p < 0.001$; one-way (**B**, **C**) and two-way (**D**) ANOVA/Bonferroni's multiple comparison test.

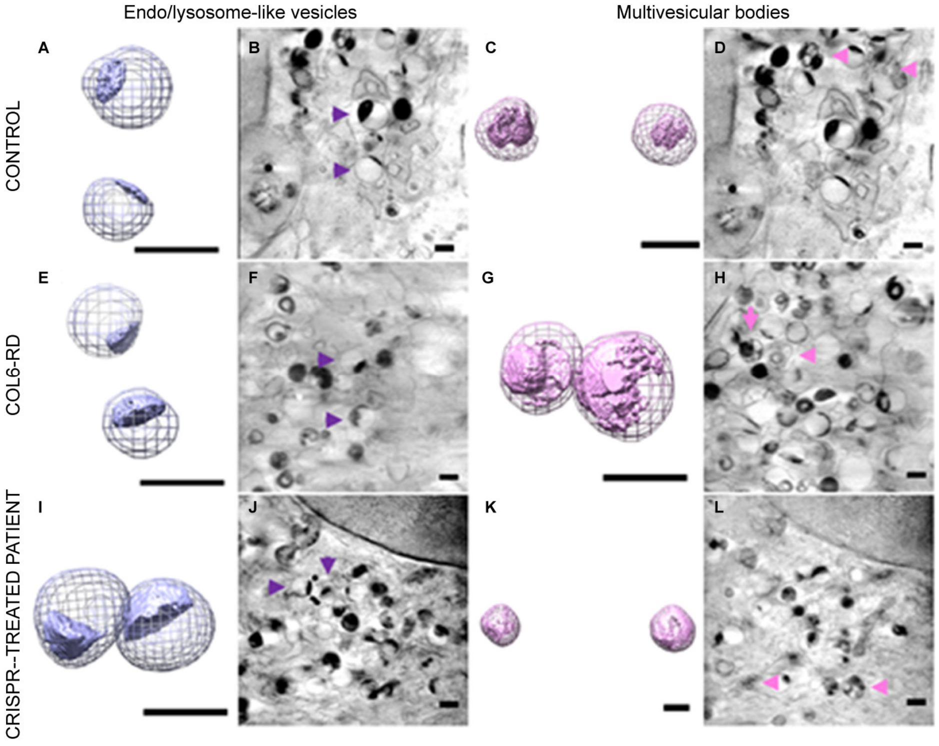

Figure S4. Morphological recognition of endosome/lysosome-like vesicles and multivesicular bodies in patient-derived fibroblasts. Three-dimensional reconstruction with threshold-based isosurface segmentation of the surface boundaries identifies the endosome/lysosome-like vesicles in violet (**A**, **E**, **J**) and multivesicular bodies

in pink (**C**, **G**, **L**). Volume slices of the tomograms from control (**B**, **D**), COL6-RD patient (**F**, **H**) and CRISPR-treated fibroblasts (**K**, **M**) where the 3D segmented structure is shown in the volume slice. Violet and pink arrows in **B**, **D**, **F**, **H**, **K**, and **M** point out the endo/lysosome-like vesicles and the multivesicular bodies respectively. Scale bar 0.5 μm.

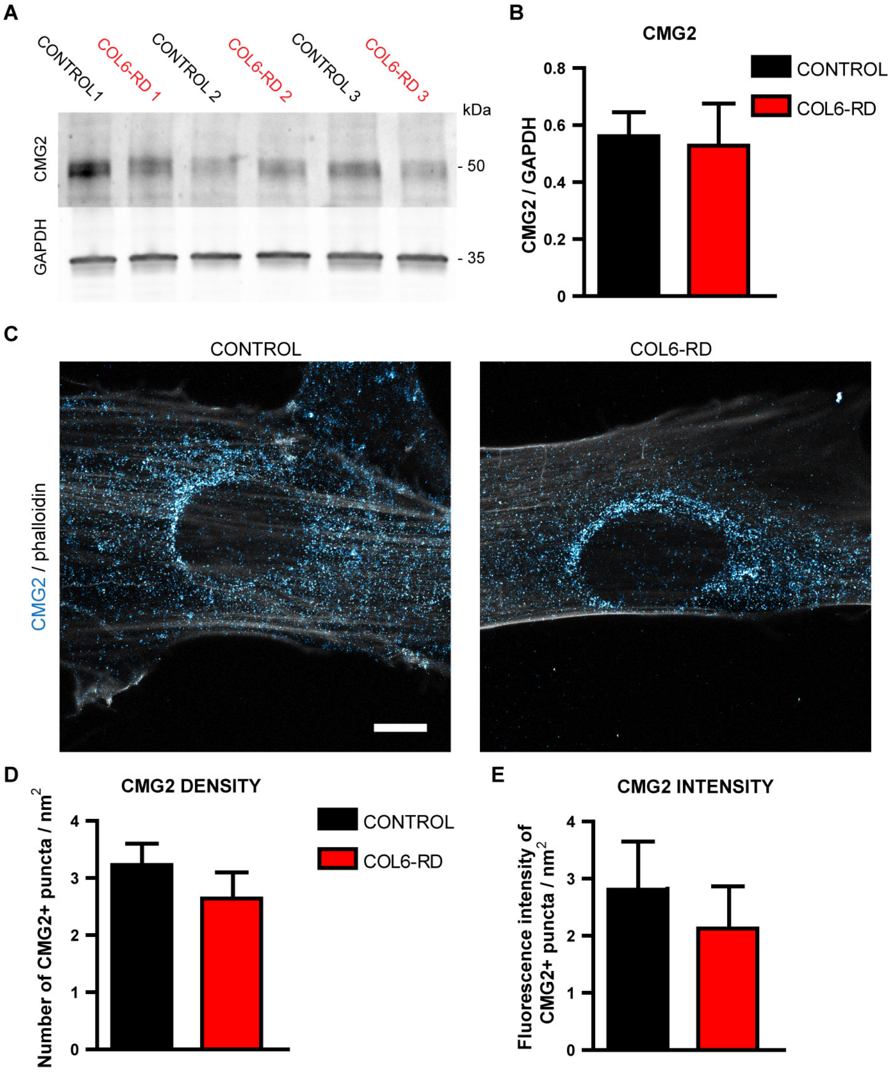

Figure S5. Patient-derived fibroblasts express CMG2 receptor. (**A**) Primary fibroblasts homogenates from controls and COL6-RD patients were subjected to Western blotting using antibodies against CMG2. The Gapdh marker was used as loading control. (**B**) Quantification of CMG2 versus Gapdh from both genotypes. (**C**) Representative STED images of patient-derived fibroblasts labelled with antibodies against phalloidin (white) and CMG2 (cyan), and relative quantification of (**D**) number of positive CMG puncta and **(E)** CMG2 fluorescence intensity from both genotypes. Data are expressed as the mean ± SEM. *n* = *3* independent primary cell preparations were used for all panels. Scale bar 5 μm.
